# Nanosecond pulsed electric fields enhance mesenchymal stem cells differentiation *via* DNMT1-regulated OCT4/NANOG gene expression

**DOI:** 10.1186/s13287-020-01821-5

**Published:** 2020-07-22

**Authors:** Kejia Li, Tong Ning, Hao Wang, Yangzi Jiang, Jue Zhang, Zigang Ge

**Affiliations:** 1 Department of Biomedical Engineering, College of Engineering, Peking University, Beijing, 100871 China; 2 Peking-Tsinghua Center for Life Sciences, Peking University, Beijing, China; 3 Institute for Tissue Engineering and Regenerative Medicine, School of Biomedical Sciences, Faculty of Medicine, The Chinese University of Hong Kong, Hong Kong, Hong Kong SAR, China; 4 School of Biomedical Sciences, Faculty of Medicine, The Chinese University of Hong Kong, Hong Kong, Hong Kong SAR, China; 5 Institute of Biomechanics and Biomedical Engineering, College of Engineering, Peking University, Beijing, China

**Keywords:** Mesenchymal stem cells, Nanosecond pulsed electric fields, Demethylation, DNMT1, Biophysical stimulation, Stem cell differentiation, Stem cell-based therapies

## Abstract

**Background:**

Multiple strategies have been proposed to promote the differentiation potential of mesenchymal stem cells (MSCs), which is the fundamental property in tissue formation and regeneration. However, these strategies are relatively inefficient that limit the application. In this study, we reported a novel and efficient strategy, nanosecond pulsed electric fields (nsPEFs) stimulation, which can enhance the trilineage differentiation potential of MSCs, and further explained the mechanism behind.

**Methods:**

We used histological staining to screen out the nsPEFs parameters that promoted the trilineage differentiation potential of MSCs, and further proved the effect of nsPEFs by detecting the functional genes. In order to explore the corresponding mechanism, we examined the expression of pluripotency genes and the methylation status of their promoters. Finally, we targeted the DNA methyltransferase which was affected by nsPEFs.

**Results:**

The trilineage differentiation of bone marrow-derived MSCs was significantly enhanced in vitro by simply pre-treating with 5 pulses of nsPEFs stimulation (energy levels as 10 ns, 20 kV/cm; 100 ns, 10 kV/cm), due to that the nsPEFs demethylated the promoters of stem cell pluripotency genes *OCT4* and *NANOG* through instantaneous downregulation of DNA methylation transferase 1 (DNMT1), thereby increasing the expression of *OCT4* and *NANOG* for up to 3 days, and created a treatment window period of stem cells.

**Conclusions:**

In summary, nsPEFs can enhance MSCs differentiation via the epigenetic regulation and could be a safe and effective strategy for future stem cell application.

## Introduction

Mesenchymal stem cells (MSCs) have been used for cell-based therapies due to their significant contribution in tissue development and regeneration [1]. Two major characteristics of MSCs, i.e., the self-renewal capacity and the differentiation potential, empower the clinical application by enlarging the cell population and contributing to the on-site neo-tissue formation. For example, bone marrow-derived MSCs, which can differentiate into osteo-, adipo-, and chondro-lineages [2], have brought positive clinical results in treating bone and cartilage defects [3, 4]. The differentiation potential of MSCs, both in vitro and in vivo, directly related to the therapeutic efficacy, depends on the tissue source (e.g., tissue derivation, health status of donor site), the cell isolation, and culture conditions [5–7]. In order to maintain the therapeutic characteristics of MSCs, many attempts have been tried, such as treatments of growth factors [8, 9] or preconditioned with hypoxia [10]. However, these methods are functioning below an effective threshold that limit the application, thus more advanced methods are needed.

Pulsed electrical stimulation (PES) has been proven to have multiple biological effects on cells for the short-term permeabilization, which mostly depends on the parameters of electric field strength (from millivolt/cm, mV/cm, to megavolt/cm, MV/cm) and stimulation duration (from nanosecond, ns, to second, s) [11]. PES-based technologies have been applied for tumor therapy, because it can induce cell apoptosis at the range of kV-MV/cm within ns-μs stimulation duration, and have been also used for electroporation for gene delivery at the range of V/cm, μs-s [11]. Nanosecond pulsed electric fields (nsPEFs), a novel technology with relatively short duration (nanoseconds, ns) and subsequent high voltages (up to kV/cm), are emerging in cell researches and have been reported to have the modulation effects on stem cells. nsPEFs can incur more comprehensive biological effects, compared with traditional pulsed electric fields (PEFs) which are above millisecond or microsecond. As the duration is shorter than the charging time of cell membrane, nsPEFs can further affect intracellular structures [11]. The biological responses of nsPEFs have been previously reported in algae cells [12], and in human cancer cell lines with the mobilization of intracellular Ca^2+^ and activation of signaling pathways [13, 14]. Besides, study has shown that nsPEFs (80 ns, 20 kV/cm, 1 Hz) may induce demethylation and activation of Suppressors of cytokine signaling (Socs) in Hela S3 cells [15]. The comprehensive and individualized reactions of cells are based on varied combinations of parameters (duration, voltage, frequency, and number of pulses) of nsPEFs, and the physical and biological properties of cells [13, 16]. nsPEFs have been studied as a possible therapeutic intervention for cancer [17–19], but little is known about their effects on regulating cell phenotype and differentiation of stem cells.

Previously, our group has found that nsPEFs could affect chondrocyte phenotype through regulating Wnt/β-catenin signaling pathway [20]. Recently, we found that nsPEFs at the levels of 10 ns at 20 kV/cm, 60 ns at 5 kV/cm, 60 ns at 10 kV/cm, 60 ns at 20 kV/cm, and 100 ns at 10 kV/cm, separately, could upregulate chondrogenic gene expression of MSCs [21]. Notably, cells can respond to physical energy epigenetically [22]; therefore, it is possible that nsPEFs have a role in epigenetic regulation of MSCs. In this study, we find that nsPEFs with specific parameters can make MSCs more susceptible to induced differentiation. In addition, we reveal that nsPEFs can downregulate DNA methylation transferase 1 (DNMT1) temporarily, and switch on the negative feedback loop between DNMT1 and OCT4/NANOG. In contrast, overexpression of DNMT1 can block the effect of nsPEFs.

## Methods

### Harvest and culture of mesenchymal stem cells

Porcine bone marrow mesenchymal stem cells (pMSCs) were harvested from three Guizhou mini-pigs (Peking University, Laboratory Animal Center, PKU-LAC) at 6 to 10 months old (approved by IACUC, PKU-LAC). Femur and tibia of the pigs were drilled before bone marrow was washed out with phosphate-buffered saline (PBS) and collected in 50 ml centrifuge tubes. Human bone marrow mesenchymal stem cells (hMSCs) were obtained from the bone marrow of three patients receiving total hip arthroplasty (male, 62 years old; male, 79 years old; female, 82 years old, from the People’s Hospital, Beijing, with IRB approval) and collected in 50 ml centrifuge tubes. After centrifuged at 1000 rpm and supernatant removed, the collected cells were suspended with medium containing 90% Dulbecco’s modified Eagle’s medium (DMEM, Gibco), 10% fetal bovine serum (FBS, Gibco), and 1% penicillin/streptomycin (PS, Amresco) and cultured at 37 °C in humidified atmosphere with 5% CO_2_. The cultured medium was changed every 3 days until the cells reached 85% of confluency. Then, they were trypsinized with 0.25% trypsin (27250–018, Invitrogen), and MSCs at passage 5 were used for all subsequent experiments.

### Application of nsPEFs

One million of MSCs were suspended in 1 mL of culture medium in gap cuvette (BTX, catalog number 45-0125, 45-0126), and were subjected to 5 pulses of nsPEFs (10 ns at 20 kV/cm, 60 ns at 5 kV/cm, 60 ns at 10 kV/cm, 60 ns at 20 kV/cm, and 100 ns at 10 kV/cm, 1 Hz). And the time interval between two pulses is 1 s [21]. MSCs not subjected to nsPEFs served as control. The nsPEFs generator was applied as previously described [23]. The voltage waveform was monitored by a Digital phosphor oscilloscope (DPO4054, Tektronix) with a probe (P6015A, Tektronix).

### qRT-PCR

Total RNA was extracted and isolated from MSCs in each stimulation condition with Trizol Reagent (New Industry) following the standard protocol and quantified with Nanodrop spectrophotometer (ND-1000, Thermo). Then, the reverse transcription reaction was performed on 500 ng of RNA with M-MLV reverse transcriptase (C28025, Sigma) and oligo (dT) (FSK-201, TOYOBO) in a PCR thermal. Quantitative real-time polymerase chain reactions (qRT-PCR) were performed in the PCR system (Pikoreal 96, Thermo) with RealMasterMix SYBR Green (FP202, Tiangen) following the manufacturer’s procedures. The expression levels of stem cell pluripotency genes and trilineage differentiation-related genes were analyzed. The primers were listed in Supplementary Table 1. The target genes of each sample were normalized to the values of *glyceraldehyde-3-phosphate dehydrogenase* (*GAPDH*) as internal control. Relative expression of each gene was expressed as fold changes by the 2^−ΔΔCt^ method. The experiment was repeated three times, with five technological repeats for each assay.

### Histology analysis of osteo-, adipo-, and chondrogenic differentiation

The treated MSCs were cultured with osteogenic induction medium, adipogenic induction medium, and chondrogenic induction medium [24] at 37 °C in humidified atmosphere with 5% CO_2_, respectively. Media was changed every 3 days. After 14 days, cell cultures were stained with Alizarin Red (AR, for osteogenic induction) and Oil Red O (ORO, for adipogenic induction) and Alcian Blue (AB, for chondrogenic induction) staining, respectively, and followed by extraction and measurement of O.D. values of AR staining at 550 nm, ORO staining at 510 nm, and AB staining at 620 nm. The experiment was repeated three times, with five technological repeats for each assay.

### Western blotting

MSCs were lysed by RIPA lysis buffer (R0020, Solarbio) with fresh protease inhibitor of 0.1% phenylmethanesulfonyl fluoride 2 h after nsPEFs, and mixed with 4× SDS loading buffer (P1015, Solarbio). The western blotting was carried out according to the manufacturer’s protocols. Rabbit polyclonal antibodies against DNMT1 (24206-1-AP, Proteintech), DNMT3a (3598, Cell Signaling), DNMT3b (orb372330, Biorbyt), and β-actin (4970S, Cell Signaling) were used in combination with secondary HRP-linked antibody of anti-rabbit IgG (7074S, Cell Signaling). The complex of the antigen and the antibody was detected with TANON 1600 Gel Imaging System (Tanon Science&Technology Co., Ltd., Shanghai), and the expression level of protein is analyzed with Tanon Gis (Tanon Science&Technology Co., Ltd., Shanghai).

### Overexpressing of DNMT1 in MSCs

For tet-on DNMT1 systems, we synthesized the coding sequence of p*DNMT1* gene from GENEWIZ by chemical method. The amplified sequence *pDNMT1* was then cloned into a pFU-tetO lentivirus backbone (19778, Addgene) linearizing with EcoR1 restriction enzyme. The FUdeltaGW-rtTA (19780) and third-generation lentiviral helper plasmid (12253, 12252, 12251) were purchased from Addgene. pFU-tetO-pDNMT1 and FUdeltaGW-rtTA were co-transfected into MSCs. Plasmids with *GFP* genes were used as control. Because there was almost no significant differences between nsPEFs with the two set parameters (10 ns at 20 kV/cm, and 100 ns at 10 kV/cm), nsPEFs of 100 ns at 10 kV/cm was used for studying the effects of downregulation of DNMT1. After stimulation by nsPEFs, doxycycline (Dox) was added to MSCs at 1 μg/ml for 2 h. The expression levels of GFP and DNMT1 were evaluated by western blotting. The primers and annealing temperatures used for PCR of *GFP* and *DNMT1* are listed in Supplementary Table 3. The experiment was repeated three times, with five technological repeats for each assay.

### Statistical analysis

Results were presented as the mean ± SD/SEM, and was normalized to the control group defined as One-way ANOVA was carried out with the least significant difference (LSD) using Prism 5.03 software (GraphPad), depending on the group numbers. The statistical significance level was set as *p* < 0.05.

## Results

### Pre-conditioning with nsPEFs enhances trilineage differentiation potential of pMSCs

**Fig. 1:**
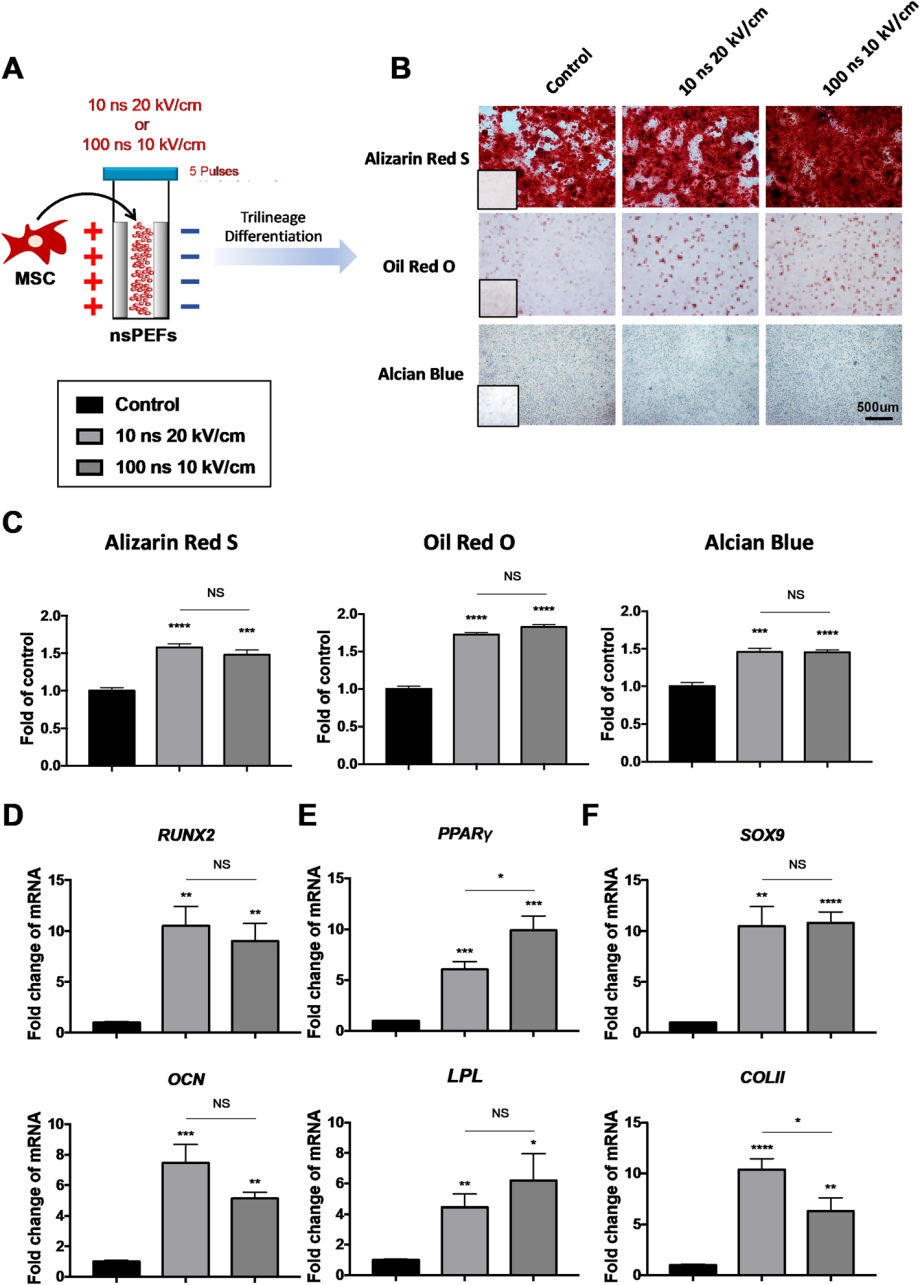
A single nsPEF treatment (5 pulses, less than 10 s) can enhance the differentiation of MSCs. **a** Schematic of MSCs stimulated by nsPEFs. One million of MSCs were suspended in culture medium in gap cuvette and were subjected to 5 pulses of nsPEFs (i.e., 10 ns at 20 kV/cm and 100 ns at 10 kV/cm). And the time interval between two pulses is 1 s. Then, the trilineage differentiation induction was carried out. **b** Alizarin Red S, Oil red O staining, and Alcian blue staining for osteogenic, adipogenic, and chondrogenic differentiation at day 14, insets show the no-staining counterparts. **c** Quantification of differentiation into osteogenic, adipogenic, and chondrogenic lineages. (3 batches of studies were tested with 3 biological donors, values are mean ± SEM from one representative batch with 5 technical repeats, one-way ANOVA, *p ≤ 0.05; **p ≤ 0.01, ***p ≤ 0.001, ****p ≤ 0.0001, NS, *p* > 0.05) **d**–**f** qRT-PCR for the mRNA levels of genes associated with trilineage differentiation (osteogenic: *RUNX2*, *OCN*; adipogenic: *PPARγ*, *LPL*; chondrogenic: *SOX9*, *COLII*) respectively at day 14. (3 batches of studies were tested with 3 biological donors, values are mean ± SEM from one representative batch with 5 technical repeats, one-way ANOVA, *p ≤ 0.05; **p ≤ 0.01, ***p ≤ 0.001, ****p ≤ 0.0001, NS, *p* > 0.05)

Stem cell properties of MSCs can be assessed by assaying the potential to differentiate along the osteogenic, adipogenic, and chondrogenic lineages [25]. In order to optimize the treatment conditions of nsPEFs, five sets of nsPEFs parameters (i.e., 10 ns at 20 kV/cm, 60 ns at 5 kV/cm, 60 ns at 10 kV/cm, 60 ns at 20 kV/cm, and 100 ns at 10 kV/cm) were firstly screened by the differentiation assays of pMSCs (Fig. 1a). We found that only nsPEFs of 10 ns at 20 kV/cm and 100 ns at 10 kV/cm had the effect of enhancing trilineage differentiation potential of pMSCs, the other three sets of parameters just enhanced one type of differentiation ability (Table 1, Fig. 1b, c).

**Table 1:** Quantification of histological staining intensity of MSCs preconditioned with five parameters of nsPEFs and differentiated to osteogenic, adipogenic, and chondrogenic lineage for 14 days

Fold *P* value	10 ns, 20 kV/cm	60 ns, 5 kV/cm	60 ns, 10 kV/cm	60 ns, 20 kV/cm	100 ns, 10 kV/cm
Osteogenic	1.58 ± 0.05****	1.34 ± 0.07*	1.15 ± 0.07 NS	1.20 ± 0.09 NS	1.48 ± 0.06***
Adipogenic	1.72 ± 0.03****	1.21 ± 0.10 NS	1.34 ± 0.10**	1.46 ± 0.10***	1.83 ± 0.03****
Chondrogenic	1.46 ± 0.05***	1.96 ± 0.17****	1.30 ± 0.08 NS	1.76 ± 0.10***	1.45 ± 0.03****

Three batches of studies were tested with 3 biological donors, values are mean ± SEM from one representative batch with 5 technical repeats, one-way ANOVA, *p < 0.05, **p < 0.01, ***p < 0.001, ****p < 0.0001, and NS, *p* > 0.05

The expression levels of differentiation genes were also evaluated at day 14. Osteogenic transcription factor *RUNX2* was upregulated by 10.53 ± 1.91- and 9.03 ± 1.77-fold by nsPEFs (10 ns at 20 kV/cm, and 100 ns at 10 kV/cm) (Fig. 1d), main regulating valves for adipogenic differentiation *PPARγ* was improved by 6.06 ± 0.78-fold (10 ns at 20 kV/cm) and 9.93 ± 1.42-fold (100 ns at 10 kV/cm) (Fig. 1e), chondrogenic transcription factor *SOX9* was increased by 10.50 ± 1.95-fold (10 ns at 20 kV/cm) and 10.82 ± 1.09-fold (100 ns at 10 kV/cm) (Fig. 1f). The expressions of other related functional genes (*OCN*, *ALP*; *LPL*, *AP2*; *COLII*, *AGG*) can be upregulated for 5–10-folds compared to the control group (Fig. 1d–f and Fig. S1A-C). Taken together, these data suggest that the biological effects of nsPEFs depend on the time and energy levels of treatment. Only two sets of parameters, i.e., 10 ns at 20 kV/cm, and 100 ns at 10 kV/cm, could enhance the differentiation potential of pMSCs.

### Optimized nsPEFs do not influence the proliferation of pMSCs

Proliferation of pMSCs was evaluated with MTT assay over 7 days after preconditioning with nsPEFs, and nsPEF treatments did not influence the proliferation of pMSCs (Fig. S2A). Moreover, cell cycle analysis and colony-forming units (CFU) assays were performed to evaluate the effects of nsPEFs. There were no significant differences in cell cycles (Fig. S2B) or CFU numbers (Fig. S2C) between nsPEF treatments and control groups. These data indicate that our optimized nsPEFs parameters do not influence the clonogenicity and proliferation of MSCs.

### nsPEFs enhance gene expressions of OCT4 and NANOG via removing the methylation of their promoters

**Fig. 2:**
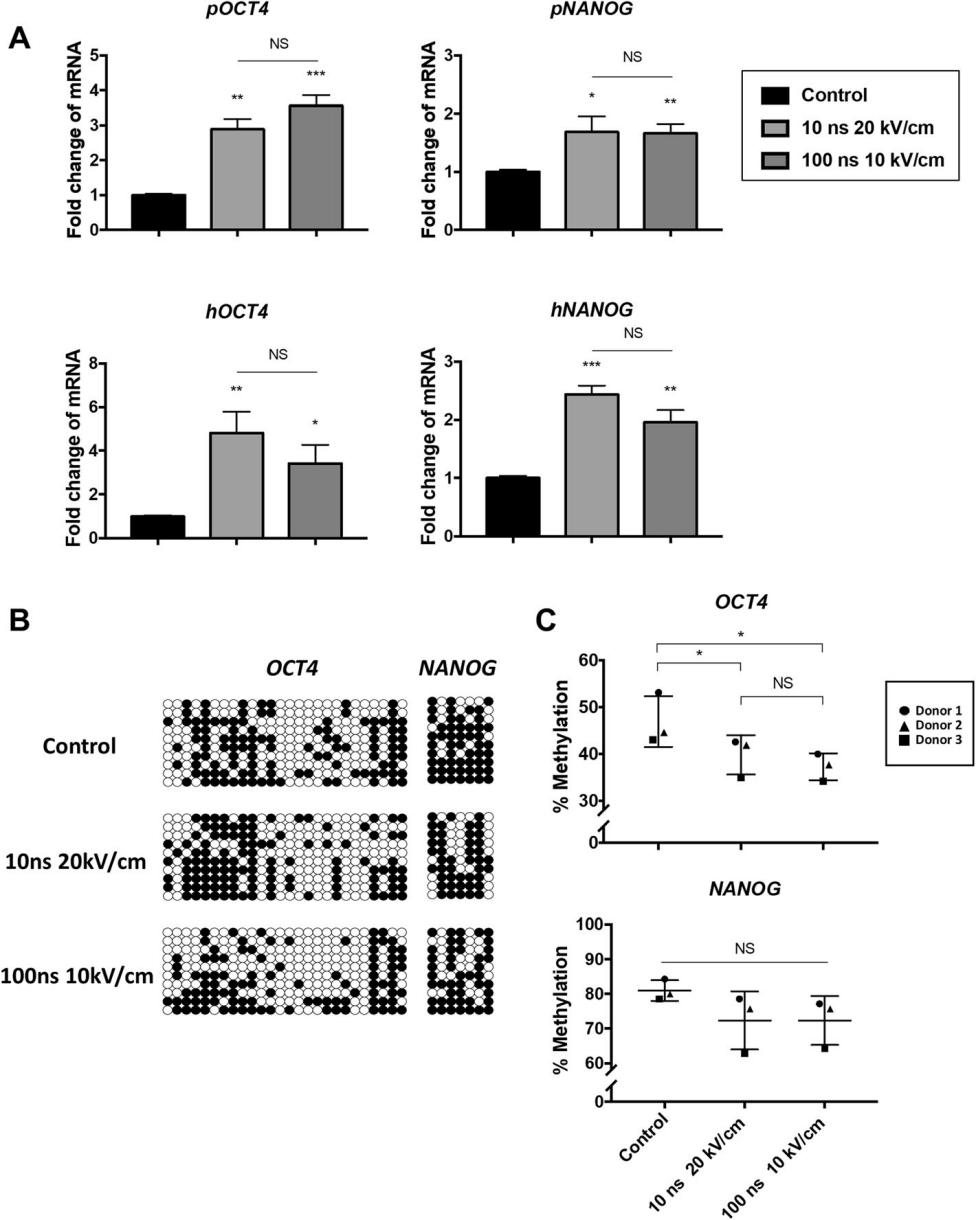
nsPEFs promote *OCT4* and *NANOG* expressions with increasing demethylation level of promoter. **a** qRT-PCR for the expressions of OCT4 and NANOG of pMSCs and hMSCs at 2 h after stimulation by nsPEFs. (3 batches of studies were tested with 3 biological donors, values are mean ± SEM from one representative batch with 5 technical repeats, one-way ANOVA. *p ≤ 0.05; **p ≤ 0.01, ***p ≤ 0.001, ****p ≤ 0.0001, NS, *p* > 0.05) **b** Bisulfite sequencing analysis of *OCT4* and *NANOG* promoter of pMSCs at 2 h after stimulation by nsPEFs. Each CpG is represented by a circle in the 50–30 orientation; each row represents the methylation state of each CpG in one bacterial clone of PCR product. White circle indicates unmethylated CpG; black circle indicates methylated CpG. **c** Percentage of CpG demethylation for each promoter. (Values are mean ± SD, *n* = 3, one-way ANOVA, *p = 0.0329, *p = 0.0171)

*OCT4* and *NANOG* are critical transcriptional factors for stem cell pluripotency [26]. To further explore the cellular molecular mechanisms of the biological effects caused by nsPEFs, the expression levels of pluripotency genes *OCT4* and *NANOG* were examined. Interestingly, an instant elevation of *OCT4* and *NANOG* was found after 2 h of nsPEF treatment both in porcine MSCs (pMSCs) and human MSCs (hMSCs) (Fig. 2a). The expression of *OCT4* increased significantly with 2.89 ± 0.30-fold changes in pMSCs (*p = 0*.*0029*), and 4.82 ± 0.97-fold in hMSCs (*p = 0*.*0044*) for 10 ns at 20 kV/cm nsPEF treatments; 3.56 ± 0.30-fold in pMSCs (*p = 0*.*001*), and 3.42 ± 0.86-fold in hMSCs (*p = 0*.*0476*) for 100 ns at 10 kV/cm of nsPEF treatments (Fig. 2a). The expression of *NANOG* was also upregulated significantly (pMSCs 1.68 ± 0.27-fold, *p = 0*.*0396* and 1.7 ± 0.16-fold, *p = 0*.*0044*; hMSCs 2.44 ± 0.15-fold, *p = 0*.*0005* and 1.96 ± 0.21-fold, *p = 0*.*0093*) in both nsPEF treatment groups (10 ns at 20 kV/cm, and 100 ns at 10 kV/cm) (Fig. 2a). We then tracked the gene expression levels of *OCT4* and *NANOG* of pMSCs at 3 days and 7 days after nsPEFs preconditioning and found that the upregulated *OCT4* subsequently decreased over 7 days (Fig. S3A and C), while the expression levels of NANOG remained the same after nsPEFs (Fig. S3B and D). In addition to the gene expression levels of *OCT4* and *NANOG*, we further examined the epigenetic modification by using bisulfite sequencing analysis. With the precondition of nsPEFs, a clearly drop was found in the methylation levels of CpG sites of *OCT4* and *NANOG* promoters, compared with non-treated pMSCs control group (Fig. 2b, c). Therefore, these data suggest that nsPEFs can directly function on MSCs by demethylating the promoter region of *OCT4* and *NANOG*.

To further investigate if the instant upregulation of pluripotency genes was a universal effect for all stem cell types, we also evaluated the *OCT4* and *NANOG* changes in human embryonic stem cells (hESCs, details are in supplementary documents) at 2 h after nsPEFs preconditioning. Interestingly, we found that only nsPEFs with parameter of 100 ns at 10 kV/cm can enhance the gene expressions of *OCT4* (4.92 ± 1.00-fold, *p = 0*.*0097*) and *NANOG* (4.63 ± 1.16-fold, *p = 0*.*0223*) of hESCs significantly, but not with 10 ns at 20 kV/cm (Fig. S3E and F).

### nsPEFs temporally decrease DNMT1 expression

**Fig. 3:**
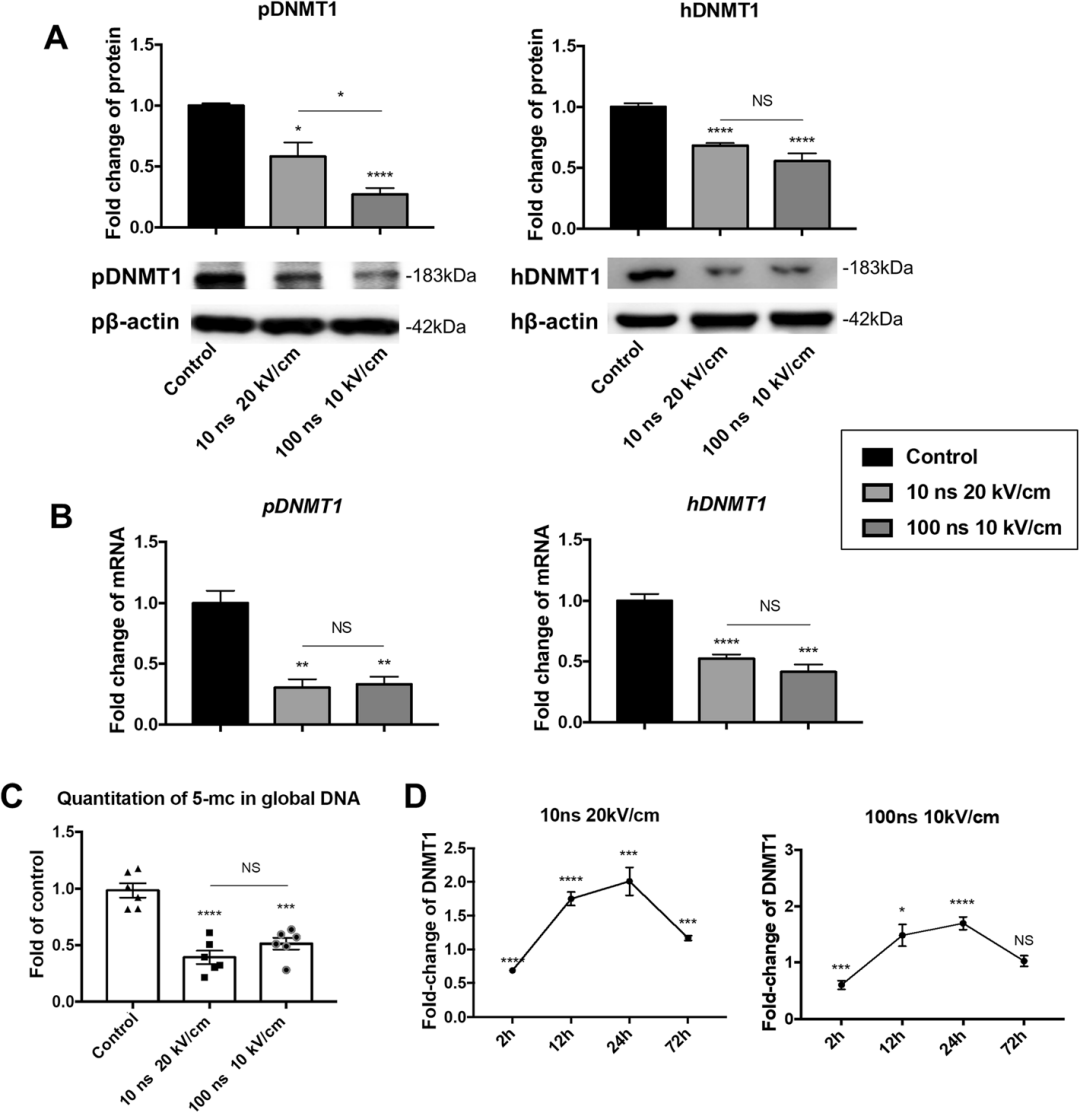
DNMT1 responds to nsPEFs with a short window of 3 days. **a** Western blot for DNMT1 protein expression level of pMSCs and hMSCs at 2 h after stimulation by nsPEFs. (3 batches of studies were tested with 3 biological donors, values are mean ± SEM from one representative batch with 5 technical repeats, one-way ANOVA, *p ≤ 0.05; **p ≤ 0.01, ***p ≤ 0.001, ****p ≤ 0.0001, NS, *p* > 0.05) **b** qRT-PCR for the expression of *DNMT1* of pMSCs and hMSCs at 2 h after stimulation by nsPEFs. (3 batches of studies were tested with 3 biological donors, values are mean ± SEM from one representative batch with 5 technical repeats, one-way ANOVA, *p ≤ 0.05; **p ≤ 0.01, ***p ≤ 0.001, ****p ≤ 0.0001, NS, *p* > 0.05) **c** The global DNA methylation level at 2 h after stimulation by nsPEFs. (*n* = 6, one-way ANOVA, ****p < 0.0001, NS = 0.1515, ***p = 0.0002) **d** Protein quantification for the expression of DNMT1 at 2, 12, 24, and 72 h after stimulation by nsPEFs. (3 batches of studies were tested with 3 biological donors, values are mean ± SEM from one representative batch with 5 technical repeats, one-way ANOVA, *p ≤ 0.05; **p ≤ 0.01, ***p ≤ 0.001, ****p ≤ 0.0001, NS, *p* > 0.05)

We next aimed to gain insights into how the hypomethylation of the *OCT4* and *NANOG* promoters was regulated by nsPEFs. DNA methylation of CpG dinucleotides is catalyzed by at least three different DNA methylation transferases (DNMTs), including DNMT1, DNMT3a, and DNMT3b. And DNMT3a and DNMT3b function primarily as de novo methyltransferases that establish DNA methylation patterns, while DNMT1 is a key enzyme that maintains methylation patterns following DNA replication [27]. The DNMTs are essential for maintaining the methylation pattern in stem cells and for regulating their self-renewal and differentiation [24, 28]. The protein expression of DNMT1 substantially dropped by 0.58 ± 0.11- and 0.27 ± 0.05-fold respectively at 2 h after nsPEF treatment (10 ns at 20 kV/cm; 100 ns at 10 kV/cm) in pMSCs, while declined to 0.69 ± 0.02- and 0.56 ± 0.06-fold in hMSCs (Fig. 3a). Gene expression of *DNMT1* decreased significantly to 0.3 ± 0.07- and 0.3 ± 0.06-fold in pMSCs, and to 0.52 ± 0.03- and 0.41 ± 0.06-fold in hMSCs (Fig. 3b). However, the levels of DNMT3a and DNMT3b did not change in both pMSCs and hMSCs (Fig. S4A and B). To confirm the function of elevated DNMT1, the 5-methylcytosine contents which reflect global DNA methylation level were measured at 2 h after nsPEFs. The global DNA methylation analysis revealed a 0.39 ± 0.06- or 0.51 ± 0.05-fold expression in nsPEFs-preconditioned groups compared with control group (Fig. 3c).

To investigate how long the effects can last, protein expression levels of DNMT1 in pMSCs at 2, 12, 24, and 72 h after nsPEFs were examined. After nsPEF treatment, the expression of DNMT1 gradually increased from a lower level at 2 h, and peaked at 24 h, which was greatly higher than control groups, and then entered the end point of a dynamic equilibrium to the levels of control groups at 72 h (Fig. 3d).

### Overexpression of DNMT1 blocks the upregulation of OCT4 and NANOG induced by nsPEFs

**Fig. 4:**
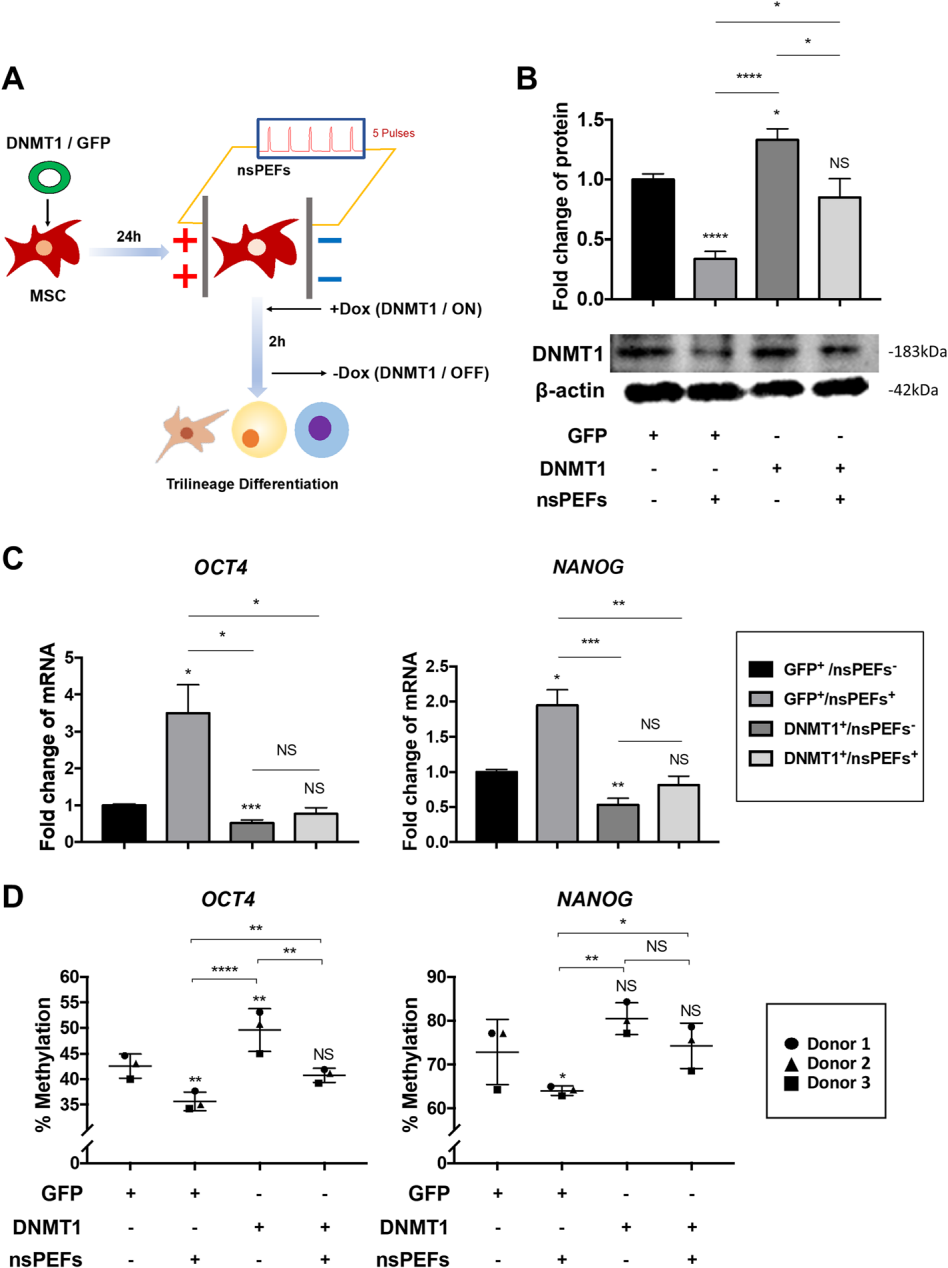
Overexpression of DNMT1 blocks demethylation caused by nsPEFs. **a** Schematic of MSCs stimulated by nsPEFs with overexpression of DNMT1. **b** Western blot for DNMT1 after pre-treated with nsPEFs with overexpression of GFP or DNMT1. (3 batches of studies were tested with 3 biological donors, values are mean ± SEM from one representative batch with 5 technical repeats, one-way ANOVA, *p ≤ 0.05; **p ≤ 0.01, ***p ≤ 0.001, ****p ≤ 0.0001, NS, *p* > 0.05) **c** Quantitative RT-PCR for the expressions of *OCT4* and *NANOG* at 2 h after stimulation by nsPEFs. (3 batches of studies were tested with 3 biological donors, values are mean ± SEM from one representative batch with 5 technical repeats, one-way ANOVA, *p ≤ 0.05; **p ≤ 0.01, ***p ≤ 0.001, ****p ≤ 0.0001, NS, *p* > 0.05) **d** Percentage of CpG demethylation for *OCT4* and *NANOG* promoter. (Values are mean ± SD, *n* = 3, one-way ANOVA, *p ≤ 0.05; **p ≤ 0.01, ***p ≤ 0.001, ****p ≤ 0.0001, NS, *p* > 0.05)

To further justify if nsPEFs-reduced DNMT1 directly affected the expressions of *OCT4* and *NANOG*, as well as the subsequent differentiation of pMSCs, we established a tet-on system to drive the DNMT1 expression in pMSCs (GFP as system control) (Fig. 4a). There were minor differences between the two sets of nsPEFs parameters (10 ns at 20 kV/cm vs. 100 ns at 10 kV/cm) in terms of the biological effects in earlier experiments. However, nsPEFs with the two parameters led to a very similar trend of regulation on DNMT1, trilineage differentiation as well as pluripotency genes of MSCs. Based on it, we assumed that nsPEFs with these two parameters regulated the cells under same or similar mechanism. And nsPEFs at the levels of 100 ns at 10 kV/cm were used in this section. Overexpression of DNMT1 by the tet-on system increased the protein expression of DNMT1 by 1.33 ± 0.09-fold (*p = 0*.*0138*), which indicated that we successfully established the DNMT1 overexpression model. Treatment of nsPEFs lowered the protein expression of DNMT1 by 0.34 ± 0.06-fold in GFP control group (Fig. 4b), which matched with the earlier results in pMSCs and hMSCs (Fig. 3a). Notably, the enhanced expression levels of *OCT4* (3.50 ± 0.77-fold, *p* = 0.0309, nsPEFs^+^ group) and *NANOG* (1.95 ± 0.22-fold, *p = 0*.*0121*, GFP^+^/nsPEFs^+^ group) were blocked by overexpression of DNMT1, and the expressions of *OCT4* and *NANOG* stayed unchanged at 2 h after nsPEF treatment (Fig. 4c, DNMT1^+^/nsPEFs^+^ group). We then evaluated the percentage of CpG demethylation of *OCT4* and *NANOG* promoters with bisulfite sequencing analysis in this DNMT1 overexpression model (Fig. 4d), and the results were consistent with the genes expression levels (Fig. 4c). Taken together, these data show that overexpression of DNMT1 can block the effects of nsPEFs on gene expressions of *OCT4* and *NANOG* in pMSCs.

### Overexpression of DNMT1 blocks the subsequent effects of nsPEFs on stem cell differentiation

**Fig. 5:**
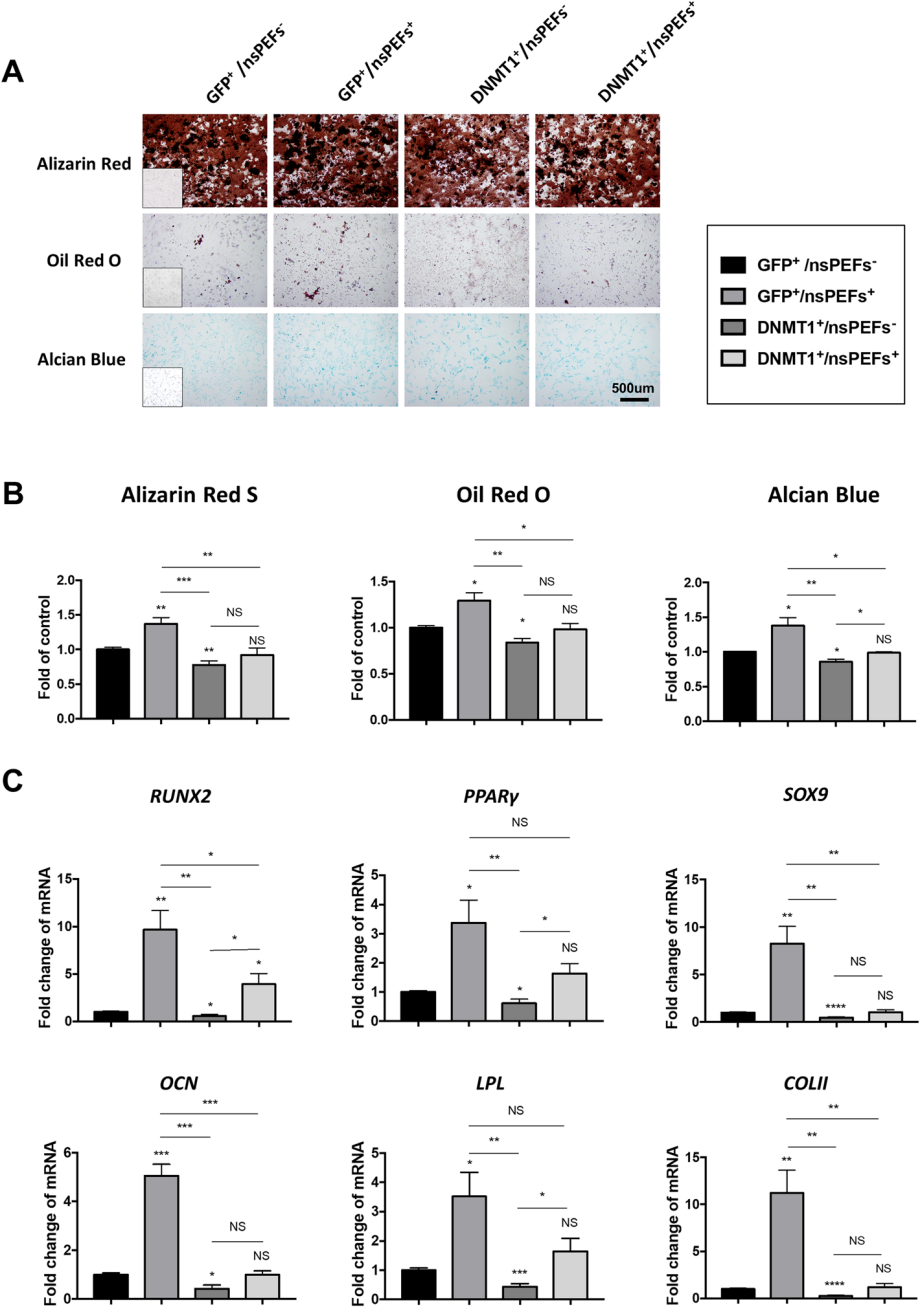
Overexpression of DNMT1 hinders the differentiation potential of MSCs caused by nsPEFs. **a** Alizarin Red S, Oil red O staining, and Alcian blue staining for osteogenic differentiation, adipogenic and chondrogenic differentiation at day 14; insets show the no-staining counterparts. **b** Quantification of differentiation into osteogenic, adipogenic, and chondrogenic lineages. (3 batches of studies were tested with 3 biological donors, values are mean ± SEM from one representative batch with 5 technical repeats, one-way ANOVA, *p ≤ 0.05; **p ≤ 0.01, ***p ≤ 0.001, ****p ≤ 0.0001, NS, *p* > 0.05) **c** MSCs were induced to undergo osteogenic, adipogenic, and chondrogenic differentiation at day 14, and qRT-PCR was performed. Trilineage differentiation-related key genes, osteogenic: *RUNX2*, *OCN*; adipogenic: *PPARγ*, *LPL*; chondrogenic: *SOX9*, *COLII* (3 batches of studies were tested with 3 biological donors, values are mean ± SEM from one representative batch with 5 technical repeats, one-way ANOVA, *p ≤ 0.05; **p ≤ 0.01, ***p ≤ 0.001, ****p ≤ 0.0001, N, *p* > 0.05)

To further investigate if DNMT1 overexpression erased the subsequent differentiation performance of MSCs enhanced by nsPEFs, both trilineage differentiation and related functional genes were evaluated (Fig. 5). Osteogenic differentiation, which was indicated by the quantification of alizarin red staining intensity (Fig. 5a, b), was increased by 1.37 ± 0.09-fold (*p = 0*.*0071*, GFP^+^/nsPEFs^+^ group) by nsPEFs (100 ns, 10 kV/cm), and decreased by 0.78 ± 0.06-fold by overexpression of DNMT1 (*p = 0*.*0068*, DNMT1^+^/nsPEFs^−^ group). Meanwhile, there was no significant difference between control group (GFP^+^/nsPEFs^−^ group) and nsPEFs stimulated DNMT1 overexpression group (*p* = 0.4912, DNMT1^+^/nsPEFs^+^ group). The differentiation performance of pMSCs into adipogenic lineage (oil-red O staining) and chondrogenic lineage (Alcian blue staining) shared the same trends as osteogenic differentiation (Fig. 5a, b). The expression levels of trilineage differentiation-related key genes (osteogenic: *RUNX2*, *OCN*; adipogenic: *PPARγ*, *LPL*; chondrogenic: *SOX9*, *COLII*) showed similar trends with the differentiation assays, that all functional genes were upregulated in GFP^+^/nsPEFs^+^ groups and had no significant change in DNMT1^+^/nsPEFs^+^ groups (Fig. 5c).

## Discussion

**Fig. 6:**
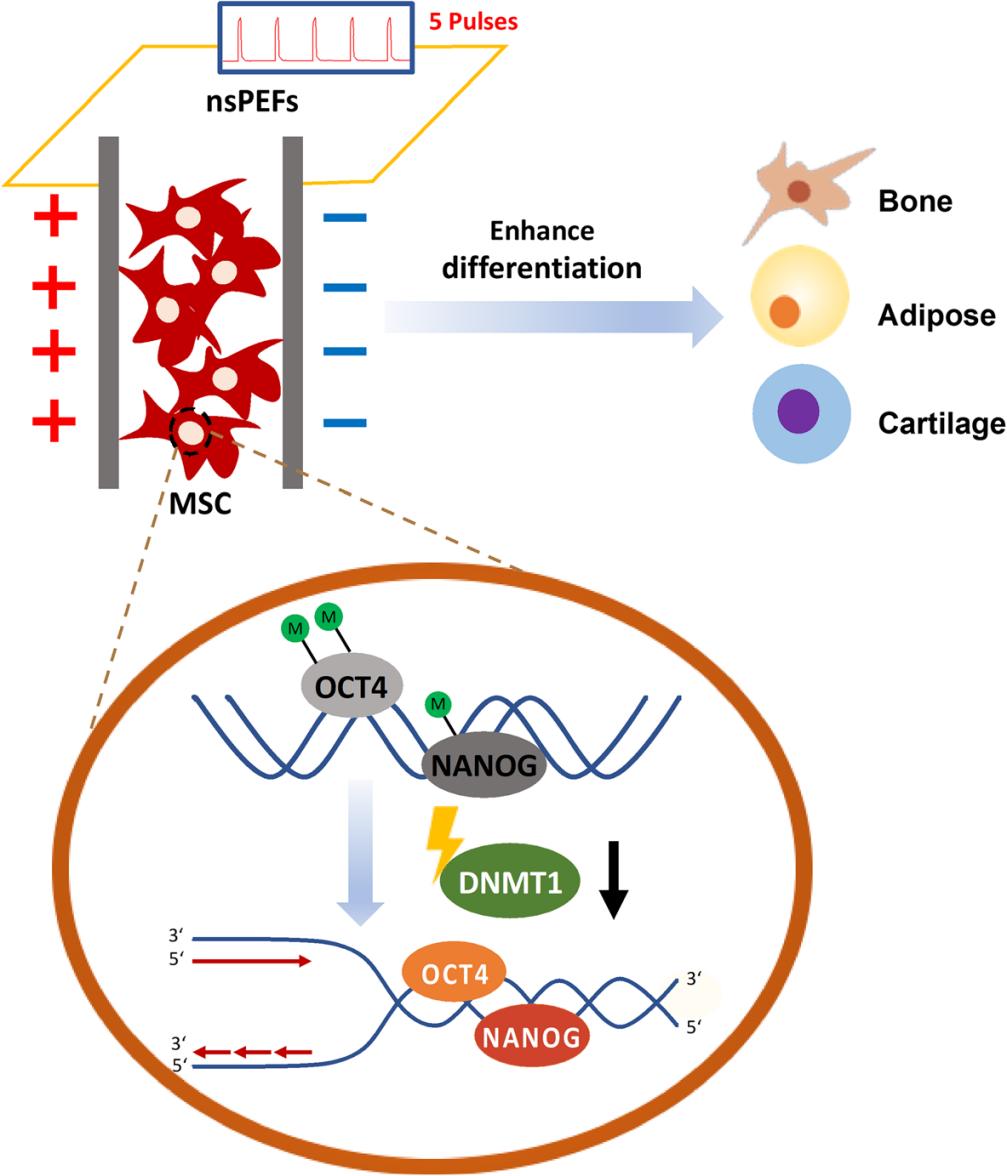
Schematic illustration of the possible molecular mechanisms induced by nsPEFs in MSCs. nsPEFs stimulation (5 pulses of electrical stimulation at levels of 10 ns at 20 kV/cm, or 100 ns at 10 kV/cm; the time interval between two pulses is 1 s) could enhance trilineage differentiation of both MSCs. In terms of the mechanism, nsPEFs could downregulate DNMT1, temporally unlock the stabilizer of DNA methylation, and lead to the elevated stem cell pluripotency gene expression of *OCT4* and *NANOG*

In this study, we discovered that a simple precondition of MSCs with nsPEFs can enhance the differentiation potential of cultured stem cells. We then investigated the cellular and molecular mechanism of this phenomenon and found that nsPEFs can remove the methylation of promoters of the pluripotency genes *OCT4* and *NANOG* temporally via downregulating the DNA methyltransferases, in particularly, the DNMT1, where the higher expressions of *OCT4* and *NANOG* were seen (Fig. 6). These nsPEF-induced epigenetic responses probably can further establish a hypersensitive phase for cell differentiation and thus performed better in all trilineage differentiation assays.

Early mechanistic studies on the biological effects of nsPEFs have indicated that the short-term high-energy stimulation can influence the intracellular membranes by electroporation and permeabilization [29], while their effects on epigenetic regulation are rarely reported. Treatment of nsPEFs with sufficient short-pulse durations and rapid rise times can induce supra-electroporation in all membranes of a cell, and extensively penetrate all membranes of organellars, supported by patch-clamp and fluorescent imaging results [29]. We here further explored the biological effects of nsPEFs and found that nsPEFs can regulate DNA modification and gene expression epigenetically.

Notably, the biological effects on cells depend on the energy level and duration of nsPEFs. As the durations decrease, the effects of the electric fields on cells shift from the cell membrane to the organelle membrane. Relatively short durations and high field strengths (hundreds kV/cm) can potentially affect the intracellular membrane while restricting total energy and narrowing down the previously broad biological effects [11]. As the two electrical parameters of nsPEFs used (10 ns at 20 kV/cm and 100 ns at 10 kV/cm) have similar voltages, it is reasonable to assume that they incur similar biological effects on MSCs. Study has shown that higher energy and longer duration (300 ns, 1.8 kV/cm) can cause cell apoptosis [30]. Indeed, it raises the safety concerns in the further application in stem cells. Our group has narrowed down a safety and effective range of nsPEFs for studying the biological effects of nsPEFs on MSCs [21]. In our previous study, we have checked the cell viability of MSCs by flow cytometry after 1 h of nsPEFs stimulation at the levels of 10 ns at 20 kV/cm and 100 ns at 10 kV/cm, which showed no difference with regular cultured cells [21]. In addition, the current study also showed that the cell proliferation, cell cycle, and colony-forming capacity of MSCs are not affected by nsPEFs at the defined levels. Our results indicated that these two sets of nsPEFs parameter (10 ns at 20 kV/cm; 100 ns at 10 kV/cm) are safe for MSCs.

Here, we found that nsPEFs can enhance stem cell differentiation through temporally fine-tuning gene expressions of *OCT4* and *NANOG*. *OCT4*, *SOX2*, and *NANOG* comprise a core transcriptional network that regulates self-renewal and pluripotency of stem cells, and are key elements for somatic cell to reprogram into iPSCs [31, 32]. These pluripotency genes are related to the differentiation potential of MSCs and can be seen as early-stage indicators and regulators of stem cell potencies [33, 34]. Many biophysical approaches can regulate the expressions of these genes and the differentiation abilities of stem cells. For instance, low-intensity pulsed ultrasound stimulation could upregulate *NANOG* expression and the subsequent osteogenic differentiation of MSCs [35]; continuous hypoxia (1% oxygen concentration) has been used to enhance and accelerate the osteogenic ability of MSCs with the upregulation of *OCT4* [36]; Overexpression of pluripotency genes can promote the differentiation of MSCs [33, 37]. In the study, we reported a similar effect of nsPEFs in regulating stem cell behaviors, that nsPEFs (10 ns at 20 kV/cm; 100 ns at 10 kV/cm) can efficiently upregulate *OCT4* and *NANOG* for 2–4-folds in human and porcine MSCs, and the precondition with 5 pulses can be done in 10 s. Our nsPEF treatment method provides a simpler and more effective way in regulating stem cells with the similar effect compared with the reported physical, chemical, or biotechnological methods, most of which request full-time consuming and complicated operation procedures.

We also found a feedback regulation between DNMT1 and *OCT4*, *NANOG* in MSCs. Previous studies have proposed that partial DNA demethylation in the *OCT4* and *NANOG* promoter regions are required for gene activation in ESCs, iPSCs, and other cell types [38–41]. *OCT4* and *NANOG* are hypomethylated in human ESCs and induced pluripotent stem cells (iPSCs) but are hypermethylated in their fibroblast derivatives [42]. DNMT1 has been shown to contribute to the methylation of *OCT4* and *NANOG* during mouse embryonic cell differentiation in vivo [43]. These research results have indicated that DNMT1 plays an important role in tissue development, and it can block the expressions of pluripotency genes and maintain a fully-differentiated stage of cells after embryo development and terminal differentiation. Here, we found that DNMT1 protein in MSCs was immediately downregulated by nsPEFs, meanwhile *OCT4* and *NANOG* gene expressions were significantly upregulated with demethylation of their promoters. These results suggested that MSCs firstly respond to nsPEFs epigenetically and genetically, and then reconstruct to a hypersensitive phase for differentiation. In addition, when DNMT1 was overexpressed, *OCT4* and *NANOG* genes remained low and unchanged, and this suggested that there was a threshold of DNMT1 in regulating *OCT4* and *NANOG* gene expressions, and certain level of DNMT1 was enough to keep the gate for *OCT4* and *NANOG* in MSCs. We also illustrated that the effects of nsPEFs on the expression of DNMT1 were a dynamic equilibrium procedure, that DNMT1 dropped at 2 h, and gradually increased and peaked to ~ 2-folds at 24 h compared to untreated control, and back to the levels of control groups at 72 h. This phenomena probably can be explained by the regulating effects of *OCT4* and *NANOG* on DNMT1, which has been reported that *OCT4* and *NANOG* can directly bind to the promoter region of DNMT1 to promote the DNMT1 expression in MSCs and fibroblasts [24, 44]. Therefore, together with our findings, we believe that there should be a feedback loop between DNMT1 and *OCT4/NANOG* in MSCs, and the regulation effect of nsPEFs on MSCs can last for 3 days. On the other hand, unaltered cell proliferation could be attributed to the balanced results of upregulated gene expression of *OCT4/NANOG* and downregulated DNMT1, for the downregulation of DNMT1 inhibits proliferation [45], and the upregulation of *OCT4*/*NANOG* promotes proliferation [26].

nsPEFs can downregulate DNMT1 temporally and enhance gene expressions of *OCT4* and *NANOG*, as well as subsequent osteo-, chondro- and adipo-genetic differentiation of MSCs, which provides us a novel and precise tool for future stem cell research. nsPEF-induced demethylation of the promoter regions of specific genes is able to achieve reversible epigenetic regulation within a treating window of 3 days. Therefore, nsPEFs-based technologies have the potential to be applied in iPSCs research to enhance the yield rate of iPSCs during reprogramming, as both the inhibition of DNMT1 [46–48] and electromagnetic fields [22] have been used to improve reprogramming efficiency, as reported previously. Despite all this, the parameters applying in iPSCs could be different from those in MSCs, because we found that only one set paraments of nsPEFs, 100 ns at 10 kV/cm, can regulate *OCT4/NANOG* in hESCs, which suggested that different cell types may need more detailed parameter segmentations. Another potential future application scenario of nsPEFs is in disease treatment, since the downregulation of DNMT1 has been reported to be able to promote the relief of the osteoarthritic symptoms in chondrocyte [49]. We have analyzed the proteomics of MSCs and found that among 3808 proteins, 59 were increased (fold change > 1.33), and 44 were downregulated (fold change < 0.75) at 2 h after nsPEF treatment, among which 6 proteins were related to epigenetic regulation (data not shown). This result suggests that more genes can be regulated via nsPEFs epigenetically. Given the unlimited parameter combinations, nsPEFs could identify multiple epigenetic targets, and regulate them either temporally or persistently, which have great potential in many bio-applications, such as in development, aging, and regeneration.

## Conclusions

This study demonstrates that nsPEFs (as levels in 10 ns at 20 kV/cm, and 100 ns at 10 kV/cm) can enhance differentiation potential of both human and porcine mesenchymal stem cells. As to the molecular mechanism, nsPEFs could temporally unlock the stabilizer of DNA methylation with downregulation of DNMT1, which lead to the upregulation of *OCT4* and *NANOG*. Taken together, nsPEFs preconditioning provides a simple and effective method to improve the differentiation potential of MSCs. And we propose that nsPEFs can further establish a hypersensitive phase for cell differentiation.

## Supplementary information

**Additional file 1 :** Supplementary Methods and Materials.

**Additional file 2 : Supplementary Tables**. **Table S1**. Primers for qRT-PCR. **Table S2**. Primers for BSP. **Table S3**. Primers for PCR.

**Additional file 3 : Figure S1**. nsPEFs Enhance trilineage differentiation of MSCs. (A-C) qRT-PCR for the expressions of functional genes of trilineage differentiation for 14 days differentiation. (3 batches of studies were tested with 3 biological donors, values are mean ± SEM from one representative batch with 5 technical repeats, one-way ANOVA, *p≤0.05; **p≤0.01, ***p≤0.001, ****p≤0.0001, NS, p>0.05).

**Additional file 4 : Figure S2**. nsPEFs have little effect on proliferation of MSCs. (A) Cell Viability based on MTT assay. (3 batches of studies were tested with 3 biological donors, values are mean ± SEM from one representative batch with 5 technical repeats, one-way ANOVA, NS, p>0.05) (B) Effect of nsPEFs on Cell cycle progression of MSCs. (3 batches of studies were tested with 3 biological donors, values are mean ± SEM from one representative batch with 5 technical repeats, one-way ANOVA). (C) Colony-forming unit assay for MSCs stimulated by nsPEFs. (D) Viable colony counts. (3 batches of studies were tested with 3 biological donors, values are mean ± SEM from one representative batch with 3 technical repeats, one-way ANOVA, NS, *p*>0.05).

**Additional file 5 : Figure S3**. nsPEFs with varied parameters incur different effects on gene expressions of pluripotency genes of stem cells. (A-D) qRT-PCR for the expression of OCT4 and NANOG of pMSCs over 7 days after stimulation by nsPEFs. (3 batches of studies were tested with 3 biological donors, values are mean ± SEM from one representative batch with 5 technical repeats, one-way ANOVA, *p≤0.05; **p≤0.01, ***p≤0.001, ****p≤0.0001, NS, p>0.05) (E and F) qRT-PCR for the expressions of OCT4 and NANOG of ESCs at 2 hours after stimulation by nsPEFs. (3 batches of studies were tested with 3 biological donors, values are mean ± SEM from one representative batch with 5 technical repeats, one-way ANOVA, *p≤0.05; **p≤0.01, ***p≤0.001, ****p≤0.0001, NS, p>0.05).

**Additional file 6 : Figure S4**. nsPEFs have no significant effect on DNMT3a/b. (A) Western blot for DNMT3a/b expression level of pMSCs at 2 hours after stimulation by nsPEFs. (3 batches of studies were tested with 3 biological donors, values are mean ± SEM from one representative batch with 5 technical repeats, one-way ANOVA, NS, p>0.05). (B) Western blot for DNMT3a/b expression level of hMSCs at 2 hours after stimulation by nsPEFs. (3 batches of studies were tested with 3 biological donors, values are mean ± SEM from one representative batch with 5 technical repeats, one-way ANOVA, NS, p>0.05).

## Data Availability

The datasets used and/or analyzed during the current study are available from the corresponding author on reasonable request.

## Abbreviations

AGG: Aggrecan
ALP: Alkaline phosphatase
COLII: Type II collagen
DMEM: Dulbecco’s modified Eagle’s medium
DNMT: DNA methyltransferase
Dox: Doxycycline
GAPDH: Glyceraldehyde-3-phosphate dehydrogenase
LPL: Lipoprotein lipase
MSCs: Mesenchymal stem cells
MTT: 3-(4,5-dimethyl-2-thiazolyl)-2,5-diphenyl-2-H-tetrazolium bromide, Thiazolyl Blue Tetrazolium Bromide
nsPEFs: Nanosecond pulsed electric fields
OCN: Osteocalcin
OCT4: Octamer-binding transcription factor 4
PPARγ: Peroxisome proliferators-activated receptor-γ
RUNX2: Runt-related transcription factor 2
Socs: Suppressors of cytokine signaling
SOX9: SRY-related high mobility group-box gene9
