# Supplementary material for: Nanosecond pulsed electric fields enhance mesenchymal stem cells differentiation via DNMT1-regulated OCT4/NANOG gene expression
Source: Stem Cell Res Ther. 2020 Jul 22;11:308. doi: 10.1186/s13287-020-01821-5 (PMC7374836; doi:10.1186/s13287-020-01821-5)
Supplement: Supplementary file 1 — Additional file 1 : Supplementary Methods and Materials. [file 13287_2020_1821_MOESM1_ESM.docx]

Supplementary Methods and Materials

*Culture of human embryonic stem cells*

H9 hESCs (WiCell Institute Inc., Madison, WI, USA) were routinely maintained on MEF feeders in the hESC medium: KnockOut Dulbecco’s modified Eagle’s medium (DMEM) culture medium supplemented with 20% (vol/vol) KnockOut serum replacement, 1% nonessential amino acids, 1 mM L-GlutaMAX-I, 0.1 mM β-mercaptoethanol, and 8 ng/ml bFGF. They were passaged with 1 mg/ml collagenase IV (Gibco17104-019, Coolaber) and seeded onto 6-well plates that had been previously coated with 0.1% gelatin solution (01-944-1B, Biological Industries).

*3-(4,5-dimethyl-2-thiazolyl)-2,5-diphenyl-2-H-tetrazolium bromide，Thiazolyl Blue Tetrazolium Bromide (MTT Assay)*

For the MTT cell proliferation assay, cells were seeded in 96-well plates at 5x10^3^ cells/well and cultured with medium containing 98% DMEM, 2% FBS, and 0.1% PS. The plates were incubated at 37ºC in humidified atmosphere with 5% CO_2_ for 1, 3 and 7 days respectively. After the specific exposure days, 20μl MTT solution (298-93-1, Coolaber) was added to each well and incubated for 3 hours in incubation. Then the solution was decanted and washed with 150μl PBS following 150μl DMSO each well. The absorbance of the samples at 540nm wavelengths was measured using a microplate spectrophotometer.

*Colony-forming unit (CFU)*

Cells were diluted in medium containing 98% DMEM, 2% FBS, and 0.1% PS, and about 20 cells were plated at per well in 6-well plates. The plates were incubated for 14d at 37°C in 5% humidified CO_2_, and wash with PBS and stain with 0.5% Crystal Violet (548-62-9, aladdin) in methanol for 5–10min at room temperature (RT). Then the plates were washed with PBS twice and visible count colonies.

*Cell Cycle Assay*

For the cell cycle assay, cells were collected, rinsed with PBS, and fixed for a minimum of 2 hours by adding 70% ice-cold ethanol at -20°C. Cells were then sequentially washed once in PBS and BD Pharmingen stain buffer (554656, BD Biosciences). Cell pellets were resuspended in 0.5 ml of BD Pharmingen PI/RNase staining buffer (550825, BD Biosciences) and incubated for 15 min at room temperature (RT), and cells were immediately analyzed using an LSRFORTESSA X-20 flow cytometer (BD Biosciences). The data were analyzed with FlowJo software.
