# Supplementary material for: Nanosecond pulsed electric fields enhance mesenchymal stem cells differentiation via DNMT1-regulated OCT4/NANOG gene expression
Source: Stem Cell Res Ther. 2020 Jul 22;11:308. doi: 10.1186/s13287-020-01821-5 (PMC7374836; doi:10.1186/s13287-020-01821-5)
Supplement: Supplementary file 2 — Additional file 2 : Supplementary Tables. Table S1. Primers for qRT-PCR. Table S2. Primers for BSP. Table S3. Primers for PCR. [file 13287_2020_1821_MOESM2_ESM.docx]

Supplementary Tables

**Table S1. Primers for qRT-PCR.**

| Gene | Primer | Tm/°C |
| --- | --- | --- |
| *pRUNX2* | F: CAGACCAGCAGCACTCCATA | 67 |
|  | R: CGGTAGCATTCTGGAAGGAG | 65 |
| *pOc* | F: CAACCCCGACT GCGACGAG | 72 |
|  | R: TTGGAGCAGCTG GGATGATGG | 70 |
| *pPPARr* | F: TCTCCGAGGGCCAAGGATTC | 69 |
|  | R: TGGGCTTCACATTCAGCAAACC | 68 |
| *pLPL* | F: GGACCTAACTTCGAGTATGCAGAA | 66 |
|  | R: CCCTCTGGTGAATGTGTGTAAGA | 66 |
| *pCOLII* | F: AGAGACCTGAACTGGGCAGA | 68 |
|  | R: TGACACGGAGTAGCACCATC | 67 |
| *pSOX9* | F: TTCATGAAGATGACCGACGA | 63 |
|  | R: GTCCAGTCGTAGCCCTTGAG | 67 |
| *pOCT4* | F: CGA AACATCACCGAGGGT | 65 |
|  | R: GCAAATGTAAAGGGCTCCTC | 64 |
| *pNANOG* | F: TCTCCGAGGGCCAAGGATTC | 69 |
|  | R: TGGGCTTCACATTCAGCAAACC | 68 |
| *pDNMT1* | F: TGGCGGGACCTACCAAACA | 69 |
|  | R: ACTTCCACGCAGGAGCAGA | 69 |
| *pGAPDH* | F: GTCATCCATGACAACTTCGG | 63 |
|  | R: GCCACAGTTTCCCAGAGG | 66 |
| *hOCT4* | F: AGCGAACCAGTATCGAGAAC | 64 |
|  | R: TTACAGAACCACACTCGGAC | 64 |
| *hNANOG* | F: TGAACCTCAGCTACAAACAG | 62 |
|  | R: TGGTGGTAGGAAGAGTAAAG | 60 |
| *hDNMT1* | F: ACCGCTTCTACTTCCTCGAGGCCTA | 73 |
|  | R: GTTGCAGTCCTCTGTGAACACTGTGG | 72 |
| *hGAPDH* | F: GTTCCAATATGATTCCACCC | 60 |
|  | R: TGAGTCCTTCCACGATACC | 63 |

**Table S2. Primers for BSP.**

| Gene | Primer | Tm/°C |
| --- | --- | --- |
| *pOCT4-U* | F: TGGAGAGCCCTGGTTTTACTG | 67 |
|  | R: ACCCCGTGGTCGGGGTG | 74 |
| *pNANOG-U* | F: GCTACAAACAGGTTAAAACCTGGTTCC | 68 |
|  | R: TTTACTAGCAGTGTATAGAAGTCCGA | 66 |
| *pOCT4-M* | F: TGGAGAGTTTTGGTTTTATTG | 58 |
|  | R: TAAAACACCAACCCCACT | 61 |
| *pNANOG-M* | F: GTTATAAATAGGTTAAAATTTGGTTTT | 55 |
|  | R: AAATAATCRTCACATATCTTCAAACT | 57 |

**Table S3. Primers for PCR.**

| Gene | Primer | Tm/°C |
| --- | --- | --- |
| *DNMT1* | F: CCGATCCAGCCTCCGCGGCCCCGGCCACCATGCCTGCCCGTACCGCCCCAGCGC | 98 |
|  | R: GATTATCGATAAGCTTGATATCGCTAGTCCTTCGTAGTCTCTTCCTCCTTGACTTTAA | 75 |
| *GFP* | F: AGACGCCTCCAGGATCCGCCACCATGGTGAGCAAGGGC | 86 |
|  | R: TTCGAACCGCGGGCCCTCTAGACTACTTGTACAGCTCGTCCATGCCGAG | 85 |
