# Supplementary figures and images for: Nanosecond pulsed electric fields enhance mesenchymal stem cells differentiation via DNMT1-regulated OCT4/NANOG gene expression
Source: Stem Cell Res Ther. 2020 Jul 22;11:308. doi: 10.1186/s13287-020-01821-5 (PMC7374836; doi:10.1186/s13287-020-01821-5)

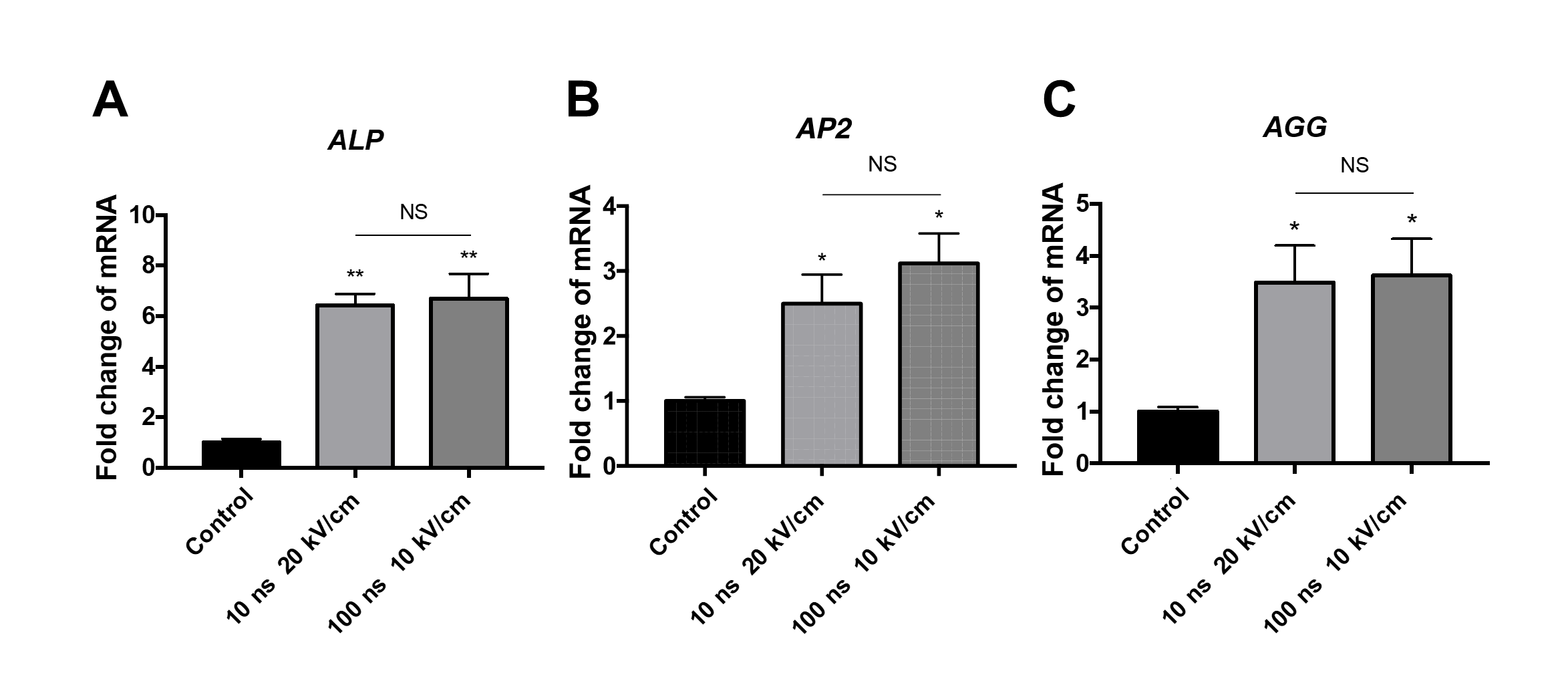

Supplement: Supplementary file 3 — Additional file 3 : Figure S1. nsPEFs Enhance trilineage differentiation of MSCs. (A-C) qRT-PCR for the expressions of functional genes of trilineage differentiation for 14 days differentiation. (3 batches of studies were tested with 3 biological donors, values are mean ± SEM from one representative batch with 5 technical repeats, one-way ANOVA, *p≤0.05; **p≤0.01, ***p≤0.001, ****p≤0.0001, NS, p>0.05). [file 13287_2020_1821_MOESM3_ESM.tif]

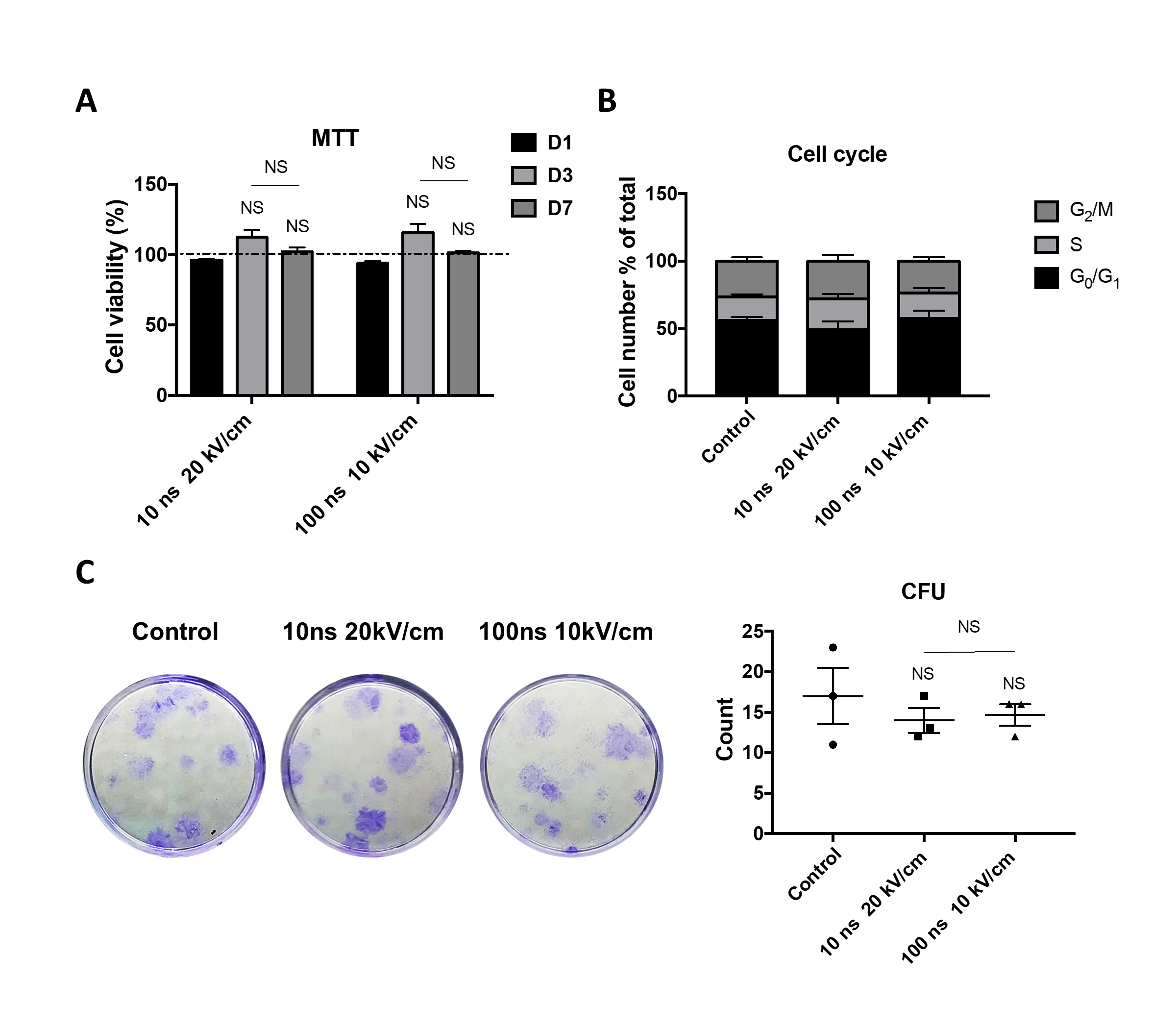

Supplement: Supplementary file 4 — Additional file 4 : Figure S2. nsPEFs have little effect on proliferation of MSCs. (A) Cell Viability based on MTT assay. (3 batches of studies were tested with 3 biological donors, values are mean ± SEM from one representative batch with 5 technical repeats, one-way ANOVA, NS, p>0.05) (B) Effect of nsPEFs on Cell cycle progression of MSCs. (3 batches of studies were tested with 3 biological donors, values are mean ± SEM from one representative batch with 5 technical repeats, one-way ANOVA). (C) Colony-forming unit assay for MSCs stimulated by nsPEFs. (D) Viable colony counts. (3 batches of studies were tested with 3 biological donors, values are mean ± SEM from one representative batch with 3 technical repeats, one-way ANOVA, NS, p>0.05). [file 13287_2020_1821_MOESM4_ESM.tif]

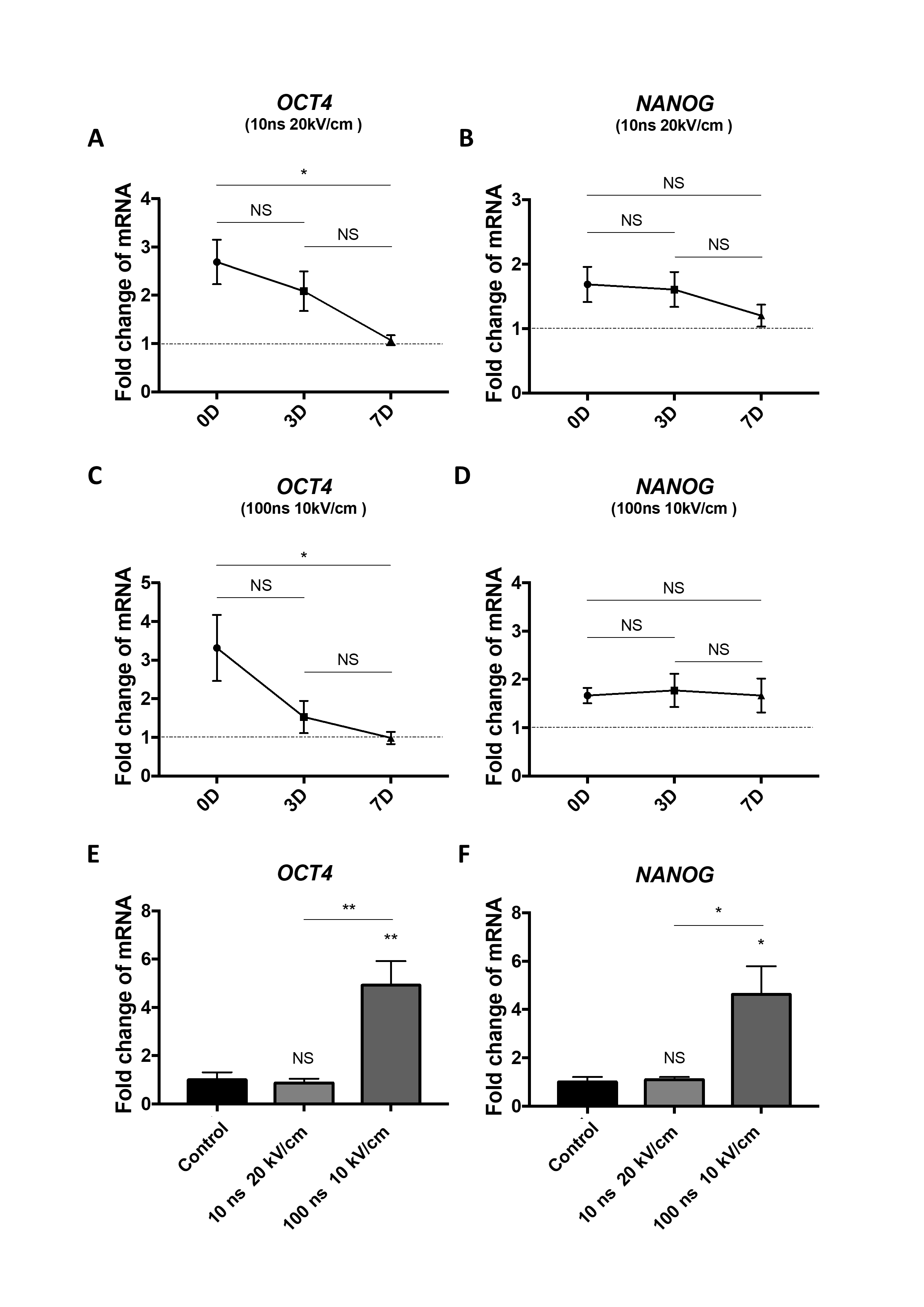

Supplement: Supplementary file 5 — Additional file 5 : Figure S3. nsPEFs with varied parameters incur different effects on gene expressions of pluripotency genes of stem cells. (A-D) qRT-PCR for the expression of OCT4 and NANOG of pMSCs over 7 days after stimulation by nsPEFs. (3 batches of studies were tested with 3 biological donors, values are mean ± SEM from one representative batch with 5 technical repeats, one-way ANOVA, *p≤0.05; **p≤0.01, ***p≤0.001, ****p≤0.0001, NS, p>0.05) (E and F) qRT-PCR for the expressions of OCT4 and NANOG of ESCs at 2 hours after stimulation by nsPEFs. (3 batches of studies were tested with 3 biological donors, values are mean ± SEM from one representative batch with 5 technical repeats, one-way ANOVA, *p≤0.05; **p≤0.01, ***p≤0.001, ****p≤0.0001, NS, p>0.05). [file 13287_2020_1821_MOESM5_ESM.tif]

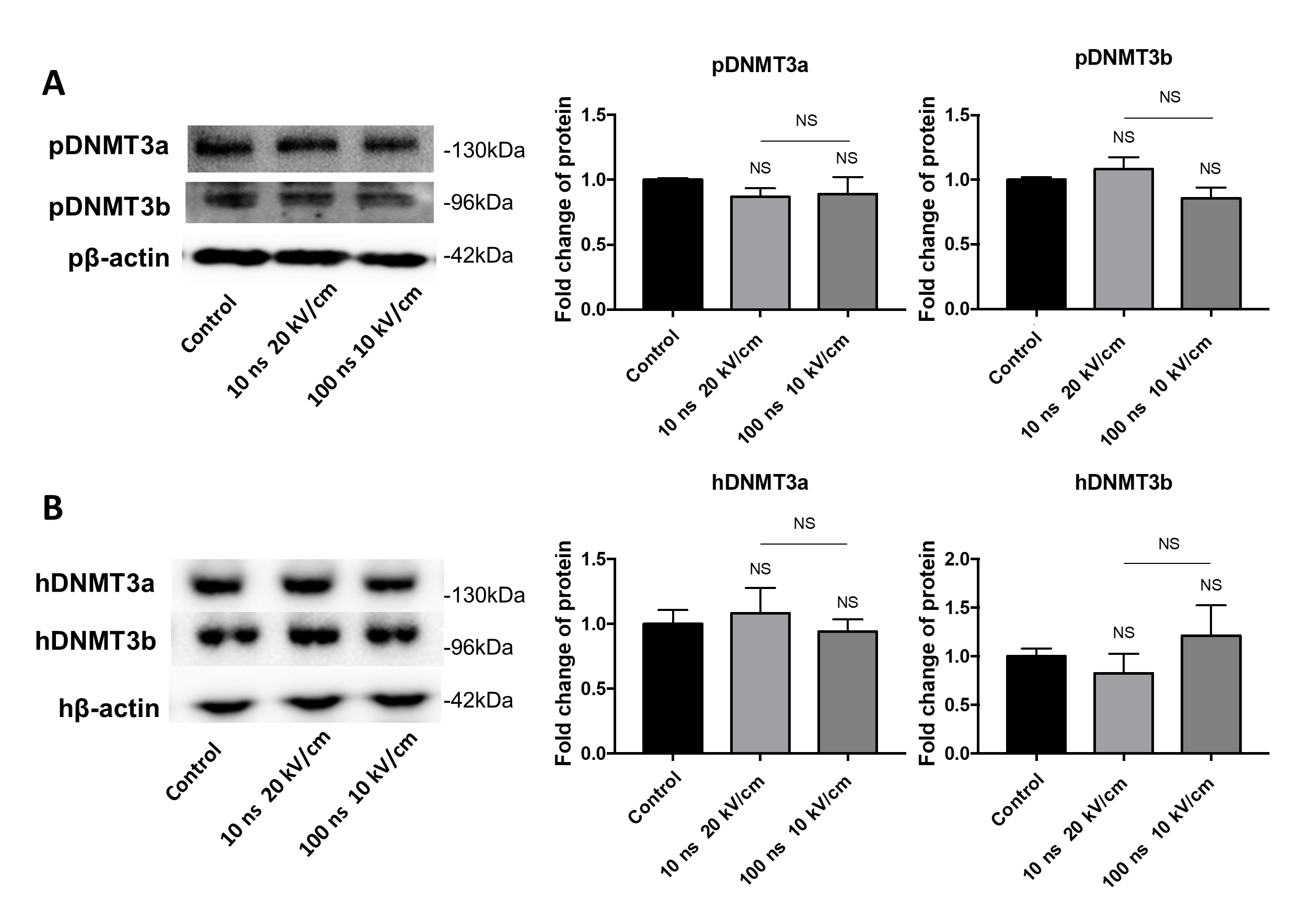

Supplement: Supplementary file 6 — Additional file 6 : Figure S4. nsPEFs have no significant effect on DNMT3a/b. (A) Western blot for DNMT3a/b expression level of pMSCs at 2 hours after stimulation by nsPEFs. (3 batches of studies were tested with 3 biological donors, values are mean ± SEM from one representative batch with 5 technical repeats, one-way ANOVA, NS, p>0.05). (B) Western blot for DNMT3a/b expression level of hMSCs at 2 hours after stimulation by nsPEFs. (3 batches of studies were tested with 3 biological donors, values are mean ± SEM from one representative batch with 5 technical repeats, one-way ANOVA, NS, p>0.05). [file 13287_2020_1821_MOESM6_ESM.tif]
